# Changes in the Thermal and Structural Properties of Polylactide and Its Composites During a Long-Term Degradation Process

**DOI:** 10.3390/polym17101326

**Published:** 2025-05-13

**Authors:** Jaroslav Cisar, Martina Pummerova, Petra Drohsler, Milan Masar, Vladimir Sedlarik

**Affiliations:** Centre of Polymer Systems, University Institute, Tomas Bata University in Zlín, Trida Tomase Bati 5678, 760-01 Zlín, Czech Republic; jcisar@utb.cz (J.C.); pvalkova@utb.cz (P.D.); masar@utb.cz (M.M.)

**Keywords:** polylactide, polymer composite, calcium carbonate, plasticizer, hydrolysis, crystallinity

## Abstract

As a polymer degrades, its structure changes, and the course of composting also affects the rate and degree of decomposition. Moreover, the potential exists for the formation of microplastics. This work focuses on the investigation of the long-term hydrolytic degradation of PLA-based composites at different temperatures (50, 55, and 60 °C, respectively). Samples were prepared on semi-industrial equipment, simulating actual production conditions. The effect of the degradation temperature on molecular weight was studied by gel permeation chromatography. Variation in the thermal properties and crystallinity of the PLA and its composites was investigated using differential scanning calorimetry and thermal gravimetric analysis. Mass loss during hydrolytic degradation was assessed using the gravimetric technique, and confirmation of microplastic residues in the hydrolyzed samples was evaluated using Fourier-transform infrared spectroscopy.

## 1. Introduction

Extensive research is being conducted to produce plastics from renewable sources, primarily in connection with polylactide (PLA). This offers several advantages, notably its favorable mechanical and optical properties, renewable origin, and ability to decompose in the natural environment. However, commercially available PLA’s thermal and mechanical resistance, along with its barrier properties, are quite poor in comparison to conventional polymers [1,2,3]. As for practical applications, packaging and consumer goods, for example, require the use of thermostable PLA-based composites [4,5,6].

Adding a suitable filler is an approach to overcoming the inherent drawbacks of PLA [1]. However, challenges arise regarding the compatibility of the filler with PLA, which can result in phase separation and embrittlement in the resulting composites. Surface modification or the use of a plasticizer (e.g., poly(ethylene glycol) (PEG)) is typically required to improve filler dispersion and strengthen the interfacial bonding between the filler and the PLA matrix [7,8,9]. Employing low-molecular-weight plasticizers is a simple and cost-effective method to enhance the mechanical properties of PLA, extending its application in the packaging field. The most discussed plasticizers for PLA composites in the literature are citrate esters, (PEG), glucose monoesters, glycerol, and oligomeric lactic acid, among others [10,11]. The incorporation of PEG into PLA has been studied extensively due to its good biocompatibility and environmentally friendly attributes.

Studies [10,11,12,13] demonstrated that PEG-based additives have the ability to significantly plasticize PLA, increasing chain mobility and lowering the glass transition and cold crystallization temperatures. Plasticized PLA exhibits greatly enhanced ductility, with elongation at break increasing more than twentyfold compared to neat PLA. However, microphase separation often occurs in PEG–PLA blends, resulting in PEG migration to the surface of the film. Chemically modifying PEG chains is advantageous for improving plasticizing efficiency, as it helps confine crystallization [10,12,14].

Calcium carbonate (CC) is a widely used low-cost filler that enhances the modulus of elasticity and increases the strength, dimensional stability, gas permeability, and physicochemical behavior of PLA. Its presence in a composition, though, could affect the inherent biodegradability and disintegration profile of PLA-based materials [2,6,8,15,16]. It also affects the rate of hydrolysis [6], with studies reporting that CC possesses a high buffering capacity, maintaining a pH of approximately 7.4 and effectively neutralizing the acidic environment caused by PLA degradation [17,18].

Another critical aspect is the end of life of PLA-based composites once they become waste [2]. PLA is often marketed as compostable; studies have focused on the disposal of such materials in industrial and domestic composting facilities [19]. Some studies claim that PLA is completely compostable [20,21], while others suggest that it degrades very slowly under suboptimal conditions [22,23,24,25]. The degradation rate of PLA-based materials depends on factors such as crystallinity, molecular weight and its distribution, morphology, the presence of plasticizers and fillers, the water diffusion rate into the polymer, type of stereoisomerism, and sample properties like surface roughness, thickness, and porosity [5,26,27,28,29].

Reports state that PLA degrades in two steps, starting with hydrolysis into low-molecular-weight oligomers, followed by microbial degradation into carbon dioxide, water, and humus. This process of hydrolysis is primarily influenced by external factors such as temperature, moisture, and pH [5,23]. Hydrolytic degradation is a frequent occurrence, induced by soluble oligomers diffusing from the surface, which leads to increased chain mobility, plasticization, chain scission, and ultimately, rapid degradation of the material [8].

A review of the literature revealed that De Santis et al. [30] and Pantani et al. [31] researched the crystallization kinetics of PLA. Their aims were to clarify the mechanism of crystallization and determine the associated morphology in molten and glassy states. Neither study addressed degradation processes, however. As mentioned above, degradation rates can be controlled by blending PLA with additives, plasticizers, and inorganic fillers (e.g., calcium carbonate (CC), barium sulfate, and mica). Although various polylactide compositions have been extensively investigated with the aim of enhancing properties and degradation processes, these studies primarily involved low-doped composites (usually up to 5%) [16,28].

Despite the existence of numerous reports on strategies for improving PLA-based materials for various applications, especially for tissue engineering [18,26,29], long-term hydrolytic degradation processes of PLA or plasticized PLA composites filled with CC fillers have not yet been described in detail. The research presented herein aimed to address this issue. It describes a comprehensive, long-term (5000 h) investigation of morphological changes that occur in PLA during degradation under abiotic conditions at various temperatures, with a focus on the influence of inorganic fillers (CC) and experimentally prepared amphiphilic block copolymers as plasticizers based on lactic acid and PEG.

## 2. Materials and Methods

### 2.1. Materials

L-lactic acid 80% (LA; Lach-Ner, Neratovice, Czech Republic), polyethylene glycol (PEG; Mn ~8000 g∙mol^−1^), the initiator Tin(II) 2-ethylhexanoate (Sn(Oct)_2_, ∼95%) (both from Sigma-Aldrich, Saint Louis, MO, USA), methanol, and acetone (p.a. grade; Penta, Prague, Czech Republic) were all utilized without further purification to synthesize the plasticizer (LA-PEG), as described below. For the filler, industrial inorganic plate-shaped calcium carbonate (CC; food-grade; particle size 3.5 µm) was purchased from Fichema (Brno, Czech Republic). PLA Ingeo 2003D (NatureWorks, Plymouth, MN, USA) was selected as the polymer matrix. This is a commercial-grade material, comprising a D% comonomer of up to 4.25 ± 0.55% (melt flow index of 6; M_n_ = 145,000; M_w_ = 235,000), for which the glass transition (T_g_) and melting point (T_m_) temperatures were 62 °C and 147 °C, respectively [30].

### 2.2. Plasticizer Synthesis

The plasticizer (LA-PEG) was synthesized by direct melt polycondensation of LA and PEG, in accordance with a procedure published in a study [12]. In brief, 500 mL of 80% LA solution was poured into a two-neck flask equipped with a Teflon stirrer and condenser. This was dehydrated at 160 °C under a reduced pressure of 20 kPa for 4 h in an oil bath. The subsequent step involved the addition of 7.5 wt.% PEG and 0.5 wt.% of Sn(Oct)_2_, upon which the reaction continued under a reduced pressure of 3 kPa for 48 h. The obtained product was then cooled, dissolved in acetone, precipitated into a solution of distilled water/methanol at the ratio of 1:1, filtered, and washed with distilled water several times. The resultant product, a powder with an Mw of 6000 g∙mol^−1^, was dried at 50 °C for 24 h.

### 2.3. Sample Preparation Procedure

A form of semi-crystalline polylactic acid (PLA Ingeo 2003D) was selected for the experiments, as it is specifically intended for the extrusion or thermoforming of packaging for fresh foodstuffs or food service ware. The sample compositions were set based on previous studies, considering the optimization of CC crystallinity initiation ability and the flexibility of the PLA matrix. The PLA granules were dried in an oven under recommended conditions (60 °C, 12 h). This was followed by the fabrication of a composite comprising PLA as the polymer matrix, the filler CaCO_3_ (CC), and the polyester-based plasticizer (LA-PEG). This was carried out on a laboratory counter-rotating twin-screw extruder (LTE26, LabTech Engineering Company Ltd., Samut Prakan, Thailand) at a screw speed of 200 rpm and processing temperatures of 110–200 °C. The resultant mixtures (Table 1) were cooled in a water bath, pelletized, and dried at 60 °C for 4 h for further preparation. A single-screw extrusion unit was subsequently employed (LE45-30/CV, LabTech Engineering Company Ltd., Samut Prakan, Thailand), equipped with a flat extrusion head for producing films 250 mm wide and approximately 0.5 mm thick. The processing parameters were set as follows: chamber zone temperatures of 150 °C, 180 °C, 195 °C, and 215 °C; flat extrusion head at 205 °C; and a screw rotation of 20 rpm. The three cooling cylinders had temperatures of 60, 55, and 40 °C; these ran at a speed of 0.7 m∙s^−1^ for gradual cooling, based on the setup of the extrusion unit. For comparison, a reference sample of the neat PLA (PLA Ingeo 2003D) matrix was prepared; the pre-dried granulate was directly extruded on a single-screw extrusion unit under conditions identical to those described above.

### 2.4. Methods

#### 2.4.1. Sampling

The potential for inhomogeneities in the processed samples was minimized by randomly removing pieces of neat PLA and composite foils for subsequent acceptance sampling; five distinct specimens were tested.

#### 2.4.2. Gel Permeation Chromatography (GPC)

Analysis was conducted to discern alterations in the molar masses of the foils prior to and following the hydrolytic degradation process. The process began with dissolution in tetrahydrofuran (THF) stabilized with butylated hydroxytoluene (BHT), followed by -filtration through a 0.45 µm syringe filter on a PL-GPC 220 chromatographic system (Agilent, Santa Clara, CA, USA) equipped with a dual detection device (a refractive index and viscometric detector). Separation was performed via 3 gel-mixed bed columns (Polymer Laboratories Ltd., Amherst, UK), comprising a PLgel-Mixed-A bed column (300 × 7.8 mm, 20 µm), a PLgel-Mixed-B bed column (300 × 7.8 mm, 10 µm), and a PLgel-Mixed-D bed column (300 × 7.8 mm, 5 µm). The mobile phase contained THF stabilized with BHT at 40 °C; the flow rate of the mobile phase was set to 1 mL∙min^−1^, and the injection volume was 100 µL. The GPC system was calibrated with polystyrene standards for molecular weights in the range of 580–6,000,000 g∙mol^−1^ (Polymer Laboratories Ltd., Amherst, UK). The results were expressed as averages of three measurements.

#### 2.4.3. Attenuated Total Reflection–Fourier Transform Infrared (ATR-FTIR) Spectroscopy

The ATR-FTIR test is a suitable method for identifying the functional groups of polymers and the molecular structures of chemicals. In this study, it was used to identify characteristic peaks in the samples at the start of the test and to compare them with the material residue (crystalline phase in powder form) after 5000 h of hydrolysis. ATR-FTIR spectra were obtained on a Nicolet iS5 instrument fitted with a diamond crystal (Thermo Fisher Scientific, Waltham, MA, USA). The resultant spectra in the wavenumber range of 400 to 4000 cm^−1^ represented averages of 64 scans at a spectral resolution of 0.8 cm^−1^.

#### 2.4.4. Thermogravimetric Analysis (TGA)

Thermogravimetric analyses of samples in platinum crucibles were carried out on a TGA Series Q500 analyzer (TA Instruments, Wilmington, NC, USA). This occurred over a temperature range of 25 °C to 1000 °C under a nitrogen atmosphere (60 mL·min^−1^) at a heating rate of 10 °C·min^−1^. The subsequent thermogravimetric (TGA) and derivative thermogravimetric curves (DTGA) expressed rates of weight loss as a function of temperature. Determination was made of the temperature at which 5% weight loss (T_5%_) occurred, as well as the peak temperature (T_p_) of the derivative of weight loss with respect to the DTGA temperature.

#### 2.4.5. Differential Scanning Calorimetry (DSC)

The thermal properties of the materials were studied by DSC on a DSC1 STARe System (Mettler Toledo, Columbus, OH, USA). Samples of approximately 5 mg were placed in aluminum pans, hermetically sealed, and inserted in the measuring cell. Measurements were taken under nitrogen (at a flow rate of 50 mL∙min^−1^) to prevent oxidative degradation at a heating/cooling rate of 10 °C∙min^−1^; the heating cycle started at 25 °C and rose to 180 °C, with subsequent cooling to 25 °C. The glass transition temperature (T_g_) was determined from the resulting thermograms, defined as the midpoint of the heat capacity increment. Temperatures for the peaks of the cold crystallization exotherm (T_cc_) and melting endotherm (T_m_), along with the enthalpies of physical transformation (ΔH_cc_ and ΔH_m_, respectively) of the PLA matrix, were evaluated from the maxima and linear integration of peaks. Analysis was performed twice for each film sample with accurate results.

#### 2.4.6. X-Ray Diffraction (XRD)

The XRD diffractometer MiniFlex600, a precision instrument, obtained X-ray diffraction measurements (RIGAKU, Tokyo, Japan) using Co Kα radiation (λ = 0.179 nm) at a voltage of 40 kV and a current of 15 mA. Diffractograms were scanned from 3° to 120° with a scan step of 0.02°at a scanning rate of 10 °/min. The XRD analysis was applied to PLA, PLA/CC, and PLA/CC/LA-PEG samples before degradation and after 5000 h of the abiotic degradation experiment at 50, 55, and 60 °C.

#### 2.4.7. Abiotic Degradation

Extruded foil samples of the PLA and composites were dried and weighed before being added to demineralized water in sterile screw flasks. Hydrolysis of samples took place at 50, 55, and 60 °C for 5000 h. Testing temperatures were selected based on the glass transition temperature of the PLA matrix, which is well known to be around 55–58 °C [9]. To determine hydrolytic degradation, pieces of the polymeric and composite films were removed at predetermined intervals (see Table S1 in Supplementary Materials) from the water medium, washed with fresh demineralized water, and dried in a vacuum oven at 45 °C and 10 mbar to constant weight. These dried specimens were then weighed, and their weight loss in percent was calculated using Equation (1):

Weight loss (%) = [(W_0_ − W_1_)/W_0_] × 100 (1)

where W_0_ is the initial weight of the sample and W_1_ is the dry weight of the sample after degradation.

#### 2.4.8. Scanning Electron Microscopy

The morphology of the powder samples after hydrolytic degradation was analyzed using a Phenom Pro unit (Phenom-World BV, Eindhoven, The Netherlands) at an electron accelerating voltage of 5 kV. No coating technique was applied.

## 3. Results and Discussion

The neat PLA foil and composites were characterized to discern their default parameters prior to undergoing hydrolytic degradation.

### 3.1. GPC

The findings for the molecular weights of the nondegraded PLA, PLA/CC, and PLA/CC/LA-PEG materials, respectively, are summarized in Table 2.

**Figure 1:**
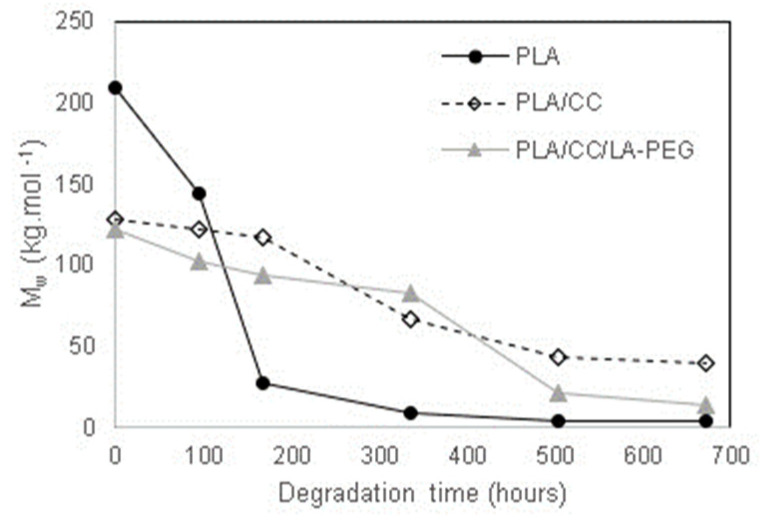
Degradation course of PLA, PLA/CC, and PLA/CC/LA-PEG samples in the first 670 h of the experiment at 60 °C.

Table 2 shows that the neat PLA exhibited higher molecular weight values compared to both composites due to the additional thermoplastic processing step applied during PLA/CC and PLA/CC/LA-PEG sample preparation. The composites originated from thermoplastically formed masterbatches; hence, the thermal history of the materials was more pronounced. In other words, the shear stress and heating that transpired in the dual extrusion processes heightened the extent of thermo-mechanical degradation in the molecular weight distribution of the PLA composites [32]. This reduction in the molar weights of the composites, compared to the neat PLA foil, was anticipated and in agreement with the literature [33,34]. No significant difference was observed between the PLA/CC and PLA/CC/LA-PEG samples in this regard, indicating that the plasticizer in the composition had no substantial effect on molecular weight. The degradation course of the samples was monitored during the degradation experiment. The effect of the abiotic degradation of PLA under various conditions was described in our previous work [35]. It was found that the degradation rate of PLA is significantly accelerated at temperatures close to or above its glass transition. In this case, the crystalline phase induced by the presence of the filler (CaCO_3_) led to the deceleration of the degradation course, expressed by the drop in GPC values (M_w_). For instance, this can be seen in Figure 1, where the degradation course in the initial stage of the experiment is shown for samples degraded at 60 °C. It is worth pointing out that GPC results are affected by the formation of the crystalline structures in PLA samples via the cold crystallization process. At elevated temperatures and in aqueous environments, the molecules in the amorphous regions of the PLA and composites tended to rearrange into a more stable crystalline state. This had an effect on both the GPC methodology itself (reduced solubility of polymer in organic solvent) and degradation kinetics (crystalline structures are more resistant to degradation (Figure 1)). This phenomenon was continuously monitored, as shown by the results of the DSC analysis. However, the GPC values of all tested samples were significantly reduced under the given degradation conditions, and after 670 h of the experiment, the samples were not suitable for GPC analysis because they approached the GPC method detection limits. Thus, GPC data for further experiment stages could not be provided.

### 3.2. ATR-FTIR Analysis

**Figure 2:**
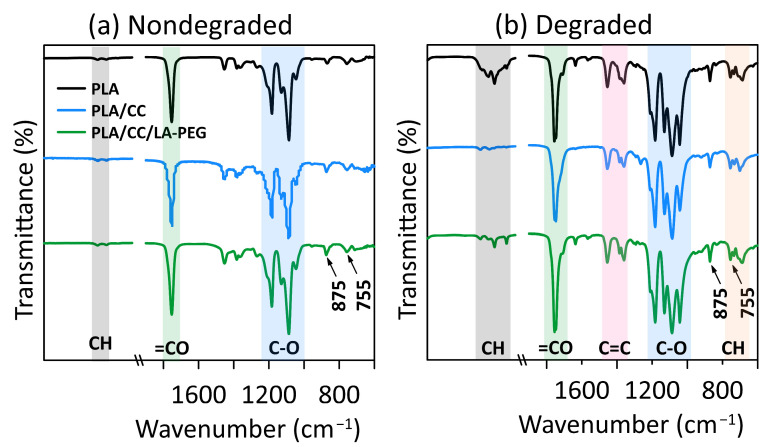
ATR-FTIR analyses of (**a**) nondegraded samples and (**b**) samples degraded for 5000 h at 60 °C.

The utility of degradable materials such as PLA depends on their resistance to aging processes. One of these is hydrolytic degradation by water, whereby water molecules diffuse into the polymer, and hydrolysis of the matrix commences. Temperature and the pH of the medium constitute crucial external factors in this context [5]. Figure 2 contains the characteristic FTIR spectra for nondegraded samples of amorphous neat PLA and composites, as well as materials following 5000 h of degradation at 60 °C. The tested samples exhibit comparable spectral patterns in both graphs, typical of a PLA matrix. The spectral bands at 1045–1205 cm^−1^ and the absorption peak at 1755 cm^−1^ correspond to C–O and C=O stretching vibrations, respectively [36]. The C=O carbonyl stretching vibration is sensitive to morphology and conformation [36,37]. Absorption bands for the bending vibrations of C=O and OH functional groups are evident at 1225 cm⁻^1^ and 1050 cm⁻^1^, respectively. The band at the lower wavenumber shows some overlap, which complicates characterization [36,37]. The spectral band at 2850–3000 cm^−1^ is linked with a CH stretching vibration, consistent with the intrinsic molecular structure of PLA. Figure 1 details the positions and shapes of the bands pertaining to the structure and characteristics of the samples. Common to all the spectra are two bands relating to the crystalline and amorphous phases of PLA occurring in the regions of 755 cm⁻^1^ and 875 cm⁻^1^. The first is assigned to the crystalline phase, while the latter relates to the amorphous phase, in agreement with the findings of the DSC analysis [37,38]. All degraded samples possess spectral bands at 680–880 cm^−1^ and 1350–1450 cm^−1^, attributed to out-of-plane CH bending vibrations and C=C stretching vibrations, respectively. This suggests the formation of unsaturated hydrocarbon groups during hydrothermal degradation, likely caused by a transformation pathway involving the conversion of lactic acid (produced by the hydrolysis of PLA) into acetic acid via hydrothermal oxidation. This pathway undergoes aldol condensation and dehydration as acidic catalysis transpires, giving rise to unsaturated ketonic acid [36].

### 3.3. TGA

**Figure 3:**
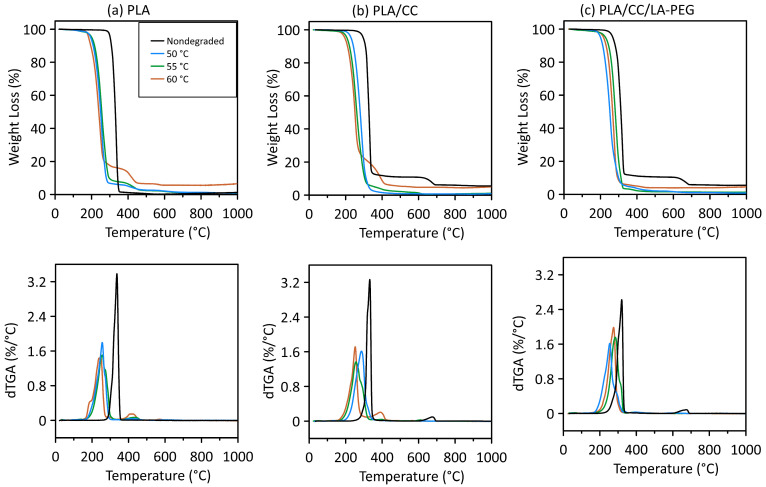
TGA and dTGA curves for samples of (**a**) PLA, (**b**) PLA/CC, and (**c**) PLA/CC/LA-PEG in nondegraded form and after 5000 h of hydrolytic degradation at 50, 55, and 60 °C.

The influence of PLA degradation on thermal stability was analyzed using TGA. Figure 3 and Table 3 present the findings for the PLA and composites in nondegraded form and after 5000 h of hydrolytic degradation at certain temperatures (50, 55, and 60 °C). The results of the thermogravimetric measurements were evaluated as follows: T_5_ (°C)—5% weight loss; T_50_ (°C)—50% weight loss; T_95_ (°C)—95% weight loss; ΔT_5–95_ (°C)—temperature difference between 5% and 95% weight loss; and T_p_ (°C)—maximum degradation temperature.

Aliphatic polyesters do not possess high thermal stability. Since PLA is such a material, it undergoes initial thermal decomposition at temperatures above 300 °C (Table 3). For neat PLA, the initial 5% weight loss (the onset of degradation) occurred at 302 °C. The decomposition temperature T_5_ for PLA/CC was 297 °C, while its filler (CaCO_3_) decomposed at 676 °C. PLA/CC/LA-PEG dropped temperature T_5_ at 268 °C, and the same filler broke down at 674 °C (see Figure 3, Table 3). This means that the presence of the CC filler leads to a decrease in the onset of the degradation of the PLA composite material. An even more pronounced effect of this type was observed for the composite with the plasticizer. Structural changes during hydrolysis (at 50, 55, and 60 °C) altered the nature of the degradation process, resulting in two-step degradation of the sample (see Figure 3). At higher temperatures, the thermal stability of PLA decreased, and the temperature range ΔT_5–95_ (°C) became larger with increasing temperature.

Rocha et al. [15] investigated the thermal stability of PBAT/PLA-based samples with 10 and 20 wt.% of CC. They concluded that adding the filler reduced the thermal stability to values lower than those of the neat polymer blend due to the catalytic effect of CC in the cleavage of ester bonds; this finding is in agreement with the data in Figure 3b,c, as well as with those in the literature [38,39].

### 3.4. DSC

The composition of materials and hydrolysis conditions affect the morphology of polymer structures in different ways. In this experiment, structural changes in the crystalline phases of the samples were analyzed in detail using DSC. PLA, as well as PLA/CC and PLA/CC/LA-PEG composites, were placed in individual glass bottles containing deionized water, according to their composition, and sealed with screw caps. These were then placed in laboratory ovens set to the desired temperature. The time of exposure amounted to 5000 h, with sampling occurring at defined intervals to determine weight loss, followed by DSC. Three temperatures were selected for testing of the given water environment, namely 50, 55, and 60 °C, thereby permitting the water molecules to freely migrate into the polymer matrix. According to Tsuji et al. [40], this would induce an autocatalytic reaction, leading to the accumulation of catalytic oligomers formed by such hydrolysis.

**Figure 4:**
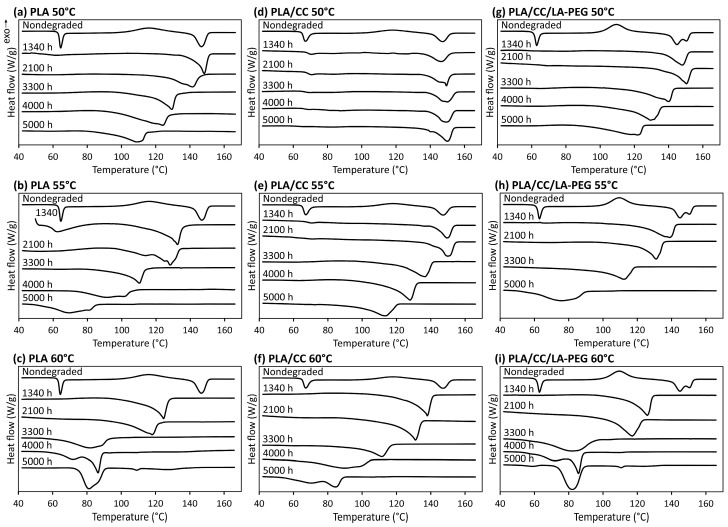
DSC curves for PLA (**a**–**c**), PLA/CC (**d**–**f**), and PLA/CC/LA-PEG (**g**–**i**) upon exposure to 50, 55, and 60 °C.

Figure 4 presents the DSC results from the first scan for the tested specimens (neat PLA, PLA/CC, and PLA/CC/LA-PEG), with detailed results in Tables S1–S3 (see Supplementary Materials). Exposure to water gradually disrupted the crystalline phase in each sample, as indicated by the broadening of endothermic melting peaks and the shift to lower melting temperatures in the graphs.

The glass transition in the amorphous phase is associated with enthalpic relaxation caused by secondary molecular relaxation [41,42]. The T_g_ values of all nondegraded specimens were in a temperature range above 55 °C. PLA exhibited a T_g_ of 62.4 °C, while PLA/CC had a slightly higher T_g_ of 64.5 °C because of the supplemented filler. The presence of the plasticizer in the PLA/CC/LA-PEG foil reduced its T_g_ to 60.1 °C. Leu et al. reported on PLA/organomontmorillonite/poly(ethylene glycol) (PEG) nanocomposites prepared using a melt intercalation technique; they described reductions induced by the PEG in the glass transition and crystallization temperatures of PLA, in accordance with the findings herein [9]. They described that the PEG in the composition raised the upper limit for moisture absorption of the PLA and decreased the diffusivity of the tested nanocomposites.

The crystalline structures of polylactide-based materials vary depending on the conditions under which crystallization takes place. The formation of such a structure for PLA and PLA/CC occurs in the solid phase (cold crystallization) and is denoted α. When PLA/CC/LA-PEG crystallizes at a temperature below 120 °C, a slightly different form of it is observed, referred to as α′. Its chain conformation and crystal formations are similar to the α structure but have a somewhat looser and less ordered structure [7,8,9]. Both α and α′ crystal structures coexist in the temperature range of 100 °C to 120 °C [8,9,10,11,12].

PLA and PLA/CC crystallize at 95 °C to 135 °C, while PLA/CC/LA-PEG crystallizes in a lower temperature range, namely at 90 °C to 130 °C (see Table S2 in the Supplementary Materials). Pantati et al. and Li et al. reported that the crystallization region of PLA typically ranges from 85 °C to 150 °C upon heating [4,13], although the fastest crystallization rate occurs between 95°C and 115 °C [14]. The latter range has also been reported by other authors to constitute the isothermal rate of crystallization [15,16,17,18]. The optimum temperature is around 110 °C for isothermal crystallization from melt [19]. Vidović et al. [16] described materials including PLA (Ingeo 3251D) composites with CC, fabricated by melt mixing in a Brabender plasticorder at 190 °C and supplemented with the given filler at concentrations of 0.1, 1, and 5 wt.%. They studied the influence of the filler on the thermal properties and crystallinity of the prepared compositions. The results showed that neat PLA prepared in this way exhibited crystallization, while the filler hindered this phenomenon in the composites and improved their thermal stability.

The melting point of PLA lies between 130 °C and 180 °C, depending on the content of L-lactide and the crystalline phase formed during crystallization [4]. The melting peak of PLA is single or double depending on the given crystalline form, the thickness of the lamellae, and the spherulites present. In addition, a double melting peak can be observed, suggesting that crystallization likely occurred within a temperature range in which both the α and α′ crystalline forms developed [20]. The neat PLA sample exhibits a single melting peak (T_m_ = 146.7 °C), as does PLA/CC (T_m_ = 147.2 °C). In contrast, PLA/CC/LA-PEG shows a double melting peak (T_m1_ = 144.8 °C and T_m2_ = 151.0 °C), as detailed in Table S3 of the Supplementary Materials.

### 3.5. Abiotic Degradation—Degradation Rate Versus Crystalline Phase Content

The composting process follows a sequential mechanism, beginning with hydrolysis, which reduces the molecular weight of PLA, followed by microbial assimilation [4]. The initial step is an autocatalytic process that generates carboxylic acids, with lactic acid acting as a catalyst for hydrolysis. These transformation pathways were confirmed by FTIR analysis. Under the influence of moisture, the ester bonds in the main polymer chain underwent hydrolysis, primarily resulting in reduced sample weight and water-soluble product formation [28]. Therefore, this research focused on hydrolytic degradation at different temperatures, with particular emphasis on changes in crystallinity within the structure. Abiotic hydrolysis experiments were conducted to determine whether temperatures near Tg accelerate PLA hydrolysis and lead to the formation of microplastic residues.

Dreier et al. confirmed that hydrolysis can occur not only at high temperatures but also at relatively mild temperatures around 60 °C and under the influence of increased humidity, e.g., at industrial composting facilities [43]. Several parameters affect its course, including prevailing environmental conditions such as water activity, temperature, pH, and time. Other associated aspects, including the degree of crystallization, molar mass, sample size and geometry, stereocomplex formation, the number of acidic end groups, and hydrophobicity, also play a crucial role [43].

In the presence of water, after a certain time, the hydrolysis of the ester groups commenced in the amorphous region of the tested materials, marking the second phase of the process during such exposure. Soluble oligomers and monomers formed by hydrolysis leached into the water, leading to a decrease in molecular weight [5]. Once the oligomers degraded enough to dissolve, the PLA polymer and its composites experienced mass loss, which was more pronounced at higher temperatures in the aqueous environment, as shown in Figure 4. A significant difference in weight loss was observed between the water temperatures; at 50 °C, it was less than at 55 °C and 60 °C.

At all the tested temperatures, the trends for decomposition were similar, with PLA/CC decomposing the slowest. The rate of weight loss for the PLA sample at 50 °C stabilized at 43.12 ± 0.17 wt.% after 4000 h. At 55 °C and 60 °C, the decomposition of PLA and PLA/CC/LA-PEG transpired in an identical manner. The literature reports that the hydrolytic degradation of PLA nanocomposites begins at the interface between the two phases formed by PLA and the appropriate filler [5].

After a certain time, hydrolytic degradation gradually leads to a decrease in the weight of the PLA sample due to the formation and diffusion of water-soluble oligomers and low-molecular-weight monomers (e.g., hydroxy acids) [43,44]. The values of the total weight loss of the neat PLA samples in the aqueous environment at 50, 55, and 60 °C were 42.4, 91.8, and 99.4 wt.%, respectively, as presented in Figure 4. For the sake of comparison, a manuscript describing how materials degraded in a composting environment was referred to; specifically, nano-reinforced PLA films that contained montmorillonite modified with an ammonium quaternary salt, CC, or silicon dioxide were tested [2]. It was found that the film samples disintegrated to a great extent, with just traces of indistinguishable residue remaining after 6–7 weeks under the given composting conditions. In contrast, it was discerned herein that, over a similar period of time, weight loss of only up to 45% transpired at temperatures typical in compost, as detailed in Figure 4. Under realistic conditions, therefore, the films degraded into microplastic residues; in this context, the values for PLA/CC dropped to 19.5, 66.8, and 96.1 wt.%, respectively. This finding agrees with those of other studies [45], in which fillers with a hydrophilic character absorbed more water than the PLA matrix, thereby complicating hydrolytic degradation and biodegradation of the material. The weight loss of PLA/CC/LA-PEG reached 56.1, 88.0, and 99.4 wt.% at the set temperatures. These results confirm that hydrolytic degradation does not necessarily result in the complete decomposition of the material [27,46].

**Figure 5:**
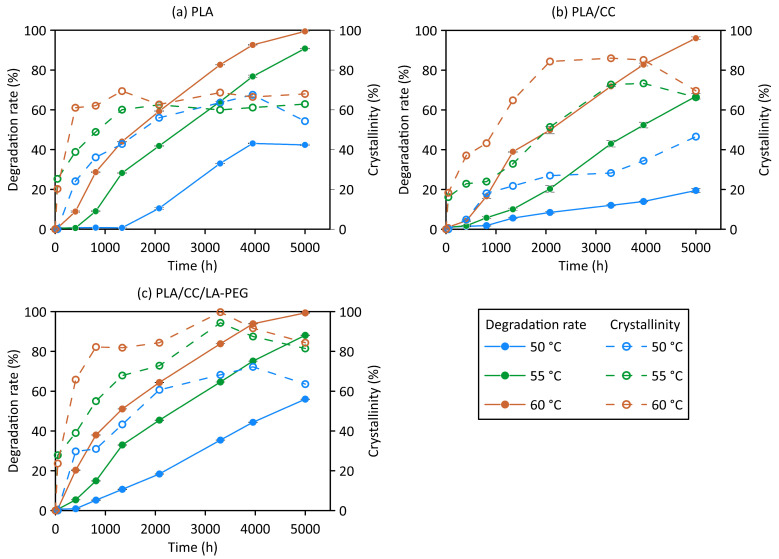
Weight loss and the crystallinity content of (**a**) PLA, (**b**) PLA/CC, and (**c**) PLA/CC/LA-PEG at different temperatures.

The hydrolytic degradation of PLA and the associated sample weight loss have been well documented. The process occurs primarily within the bulk of the material, rather than on its surface. The cleavage of polymer chains during hydrolysis occurs preferentially in the amorphous regions, increasing the overall crystallinity of the polymer. Consequently, hydrolysis is significantly faster in these regions than in semi-crystalline ones [46]. Macromolecular polymer chains in crystalline regions are more resistant to hydrolysis than those in amorphous regions, as water molecules have very limited access to the chains inside the rigid crystalline areas [47]. This increases the content of the crystalline phase in the sample. Once a certain degree of crystallinity is reached, a plateau appears, followed by a tendency for a decrease (Figure 5) [48,49]. Slow hydrolysis of the chains at the surface of the crystalline regions then gradually commences, leading to their degradation. By the end of the experiment, only a powdery residue remained from the original film samples after they had been dried. The degree of crystallinity, calculated from the mean melting enthalpy value of each group, was enhanced by nucleation, a phenomenon caused by supplementing the PLA matrix with additives, in agreement with the literature [26].

Water diffusion into the polymer matrix, effectively acting as a plasticizer, in combination with temperature, triggered a rapid rise in the crystalline phase content in the neat PLA, PLA/CC, and PLA/CC/LA-PEG samples at the beginning of the experiment, especially in the amorphous materials (see Figure 4). Water penetrates the amorphous phase, and at elevated temperatures (above T_g_) in an aqueous environment, PLA polymer molecules and PLA in composite materials tend to rearrange into a more stable crystalline state—a process known as PLA recrystallization (see the DSC curves in Figure 4). Additionally, the initial crystallization rate of PLA and its composites increases with rising temperature.

### 3.6. X-Ray Diffraction (XRD)

**Figure 6:**
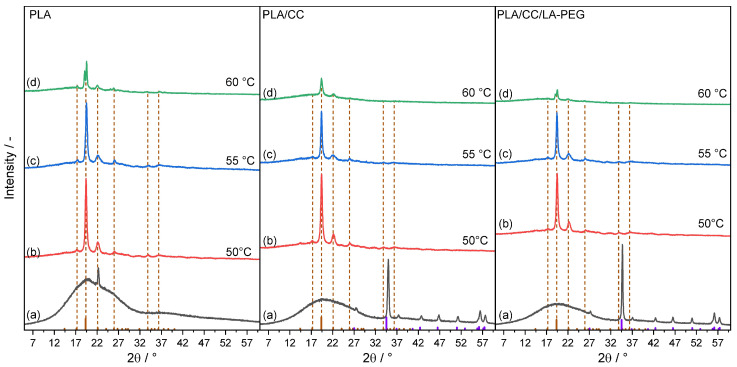
XRD patterns of PLA, PLA/CC, and PLA/CC/LA-PEG samples before (**a**) and after 5000 h of degradation at (**b**) 50, (**c**) 55, and (**d**) 60 °C.

A long-term comprehensive experiment to study the effect of temperature on the hydrolytic degradation of the structural properties of PLA, PLA/CC, and PLA/CC/LA-PEG samples in an aqueous environment was conducted (Figure 5). XRD diffractograms were acquired to elucidate the characteristics of the residual material present following the degradation process, as illustrated in Figure 6. The 2θ values of the XRD pattern for all samples (specifically from 2 to 4, representing the crystalline phase structures) align with the standard values of polylactic acid (PLA) for the peaks observed at 17.2°, 19.3°, 22.1°, 26.0°, 33.8°, and 36.3°, which are indexed to the diffraction planes (104), (200), (203), (211), (310), and (217), respectively. In composite materials, calcium carbonate (CaCO_3_) as a filler is evident (PLA/CC and PLA/CC/LA-PEG). The influence of temperature is apparent and may accelerate the degradation process due to the enhanced hydrolysis of PLA and the breakdown of ester bonds, resulting in diminished molecular weights [50]. Furthermore, the degradation of PLA generates acidic degradation products, such as lactic acid, which can potentially compromise the structural integrity of the foils or fillers. As demonstrated in the presented diffractograms, the filler may undergo separation or degradation in an acidic environment. Additionally, as indicated by differential scanning calorimetry (DSC), the crystalline fraction of PLA is liberated from the prepared films during the process. Based on the observed decrease in intensity and quantity of post-degradation products, it can be posited that elevated temperatures facilitate the subsequent degradation of these crystalline moieties.

### 3.7. Scanning Electron Microscopy

**Figure 7:**
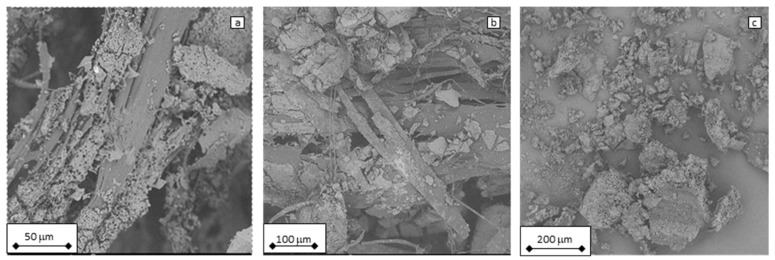
SEM micrographs of PLA sample after 5000 h of hydrolysis at (**a**) 50, (**b**) 55, and (**c**) 60 °C.

SEM micrographs of the PLA samples after 5000 h of hydrolysis at 50 (a), 55 (b), and 60 °C (c) are shown in Figure 7. It can be seen that the fragmentation of the samples is more intensive with increased degradation temperatures applied during the experiment. This is related to the abovementioned results, indicating a significantly higher PLA degradation rate above its glass transition temperature.

**Figure 8:**
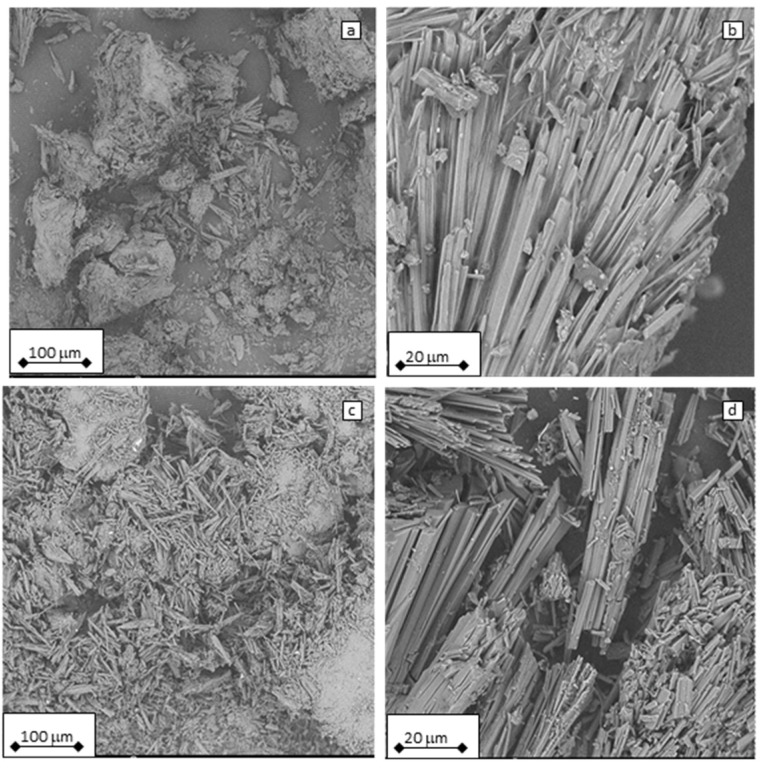
SEM micrographs of (**a**,**b**) PLA/CC and (**c**,**d**) PLA/CC/LA-PEG samples after 5000 h of hydrolysis at 60 °C.

Fragments of the PLA/CC (a, b) and PLA/CC/LA-PEG (c, d) samples (5000 h, 60 °C) can be found in Figure 8. The needle-like residuals of CC are clearly visible, while PLA fragments are not visible. Obvious microparticles of PLA were found, even after 5000 h of PLA degradation above its glass transition temperature. This raises further questions about the formation of microplastics and their environmental fate in relation to the use of a PLA matrix, which is considered biodegradable. In addition, inorganic microparticle formation is present in the case of PLA composites by means of fillers released from the degraded polymer matrix.

## 4. Conclusions

PLA and its composites are among the most frequently used bioplastic-based materials. Despite broad research conducted on its degradation process, a detailed analysis of its fate after longer time periods has not been reported. The findings reported in this work are important for the evaluation of the environmental impact of PLA and its composites after their lifetime, as well as after the period defined by standards for biodegradation determination.

A detailed study was conducted on the morphological changes induced by the abiotic degradation of the PLA, PLA/CaCO_3_, and PLA/CaCO_3_/LA-PEG systems at temperatures of 50, 55, and 60 °C over a period of 5000 h. The findings showed that the degradation process in the aqueous medium was significantly influenced by the morphology and volume fractions of the crystalline and amorphous regions. Hydrolytic degradation accelerated with increasing temperature (50 °C < 55 °C < 60 °C). Spectroscopy indicated the formation of unsaturated hydrocarbon groups, while DSC analysis revealed the gradual disruption of the crystalline phase, as evidenced by the broadening of endothermic melting peaks and shifts in melting temperatures to lower values during hydrothermal degradation. As hydrolysis progressed, the foil samples fragmented into smaller particles. By the end of the experiment (5000 h), the dried particle size was estimated to be less than 0.5 mm, so it was classified as microplastic. These findings may prompt further research aimed at describing PLA microplastics and inorganic particles released from degraded polymer matrix interactions with the environment.

## Figures and Tables

**Table 1:** Sample compositions.

Name	PLA Content (wt.%)	CaCO_3_ Content (wt.%)	Plasticizer Content (wt.%)
PLA	100	-	-
PLA/CC	90	10	-
PLA/CC/LA-PEG	86	10	4

**Table 2:** GPC results for the nondegraded samples.

Name	M_n_ (g·mol^−1^)	M_w_ (g·mol^−1^)	M_p_ (g·mol^−1^)	PDI (-)
PLA	103,000 ± 9000	209,000 ± 5000	215,000 ± 1000	1.89 ± 0.37
PLA/CC	63,000 ± 5000	128,000 ± 2000	147,000 ± 4000	2.05 ± 0.16
PLA/CC/LA-PEG	64,000 ± 4000	122,000 ± 3000	143,000 ± 3000	1.91 ± 0.07

**Table 3:** TGA analysis of samples in nondegraded form and after 5000 h of hydrolytic degradation at 50, 55, and 60 °C.

	T_5_ (°C)	T_50_ (°C)	T_95_ (°C)	Δ T_5–95_ (°C)	T_p_ (°C)
**PLA**					
Nondegraded	301.8	330.5	346.8	16.3	336.3
50 °C	221.1	276.9	357.6	80.7	266.3
55 °C	206.3	263.7	373.9	110.2	260.8
60 °C	183.8	239.7	598.9	359.2	246.7
**PLA/CC**					
Nondegraded	297.1	328.1	-	-	332.8 676.4
50 °C	229.6	280.0	326.6	46.6	286.7
55 °C	207.9	260.6	360.7	100.1	256.0 395.6
60 °C	195.5	250.6	555.5	305.9	252.1 391.0
**PLA/CC/LA-PEG**					
Nondegraded	267.6	311.8	-	-	320.0 674.2
50 °C	200.1	251.3	344.4	93.1	253.7
55 °C	203.2	256.8	388.7	131.9	255.2
60 °C	216.4	266.8	402.6	135.8	276.0

## Data Availability

The original contributions presented in this study are included in the article. Further inquiries can be directed to the corresponding author.

## Supplementary Materials

The following supporting information can be downloaded at: https://www.mdpi.com/article/10.3390/polym17101326/s1, Table S1: DSC results of glass transition temperature (T_g_) of samples at 50, 55, and 60 °C; Table S2: DSC results of crystallization behavior (T_c_ and ΔH_c_) of samples; Table S3: DSC results of melting behavior (T_m_ and ΔH_m_) of samples.
