# Supplementary material for: Changes in the Thermal and Structural Properties of Polylactide and Its Composites During a Long-Term Degradation Process
Source: Polymers (Basel). 2025 May 13;17(10):1326. doi: 10.3390/polym17101326 (PMC12114928; doi:10.3390/polym17101326)
Supplement: Supplementary file 1 [file polymers-17-01326-s001.zip › polymers-3584797-supplementary.pdf]

polymers

MDPI

Supplementary Materials

Changes in the Thermal and Structural Properties of Polylactide and Its Composites During a Long-Term Degradation Process

Jaroslav Cisar, Martina Pummerova *, Petra Drohsler, Milan Masar and Vladimir Sedlarik *

Centre of Polymer Systems, University Institute, Tomas Bata University in Zlín, Trida Tomase Bati 5678, 760-01 Zlín, Czech Republic
* Correspondence: pummerova@utb.cz (M.P.); sedlarik@utb.cz (V.S.); Tel.: +420-576-031-740 (M.P.); +420-576-038-013 (V.S.)

Table S1. DSC results of glass transition temperature (T_g) of samples at 50, 55, and 60 °C.

Time (h)	PLA	PLA/CC	PLA/CC/P
0	62.4	64.5	60.2
		**50 °C**	
46	61.7	64.4	62.8
400	63.3	64.2	64.2
810	62.5	65.1	63.0
1340	-	68.0	-
2100	-	69.6	-
3300	-	-	-
4000	-	-	-
5000	-	-	-
		**55 °C**	
46	63.7	64.4	62.1
400	-	65.1	62.7
810	63.0	64.2	63.5
1340	-	68.2	-
2100	-	68.0	-
3300	-	-	-
4000	-	-	-
5000	-	-	-
		**60 °C**	
46	63.2	64.4	64.3
400	-	65.5	64.3
810	-	-	-
1340	-	-	-
2100	-	-	-
3300	-	-	-
4000	-	-	-
5000	-	-	-

Table S2. DSC results of crystallization behavior (T_c and ΔH_c) of samples.

	PLA		PLA/CC		PLA/CC/P	
**Time (h)**	**T_c (°C)**	**ΔH_c ($J\cdot g^{-1}$)**	**T_c (°C)**	**ΔH_c ($J\cdot g^{-1}$)**	**T_c (°C)**	**ΔH_c ($J\cdot g^{-1}$)**
0	115.9	25.72	117.6	17.54	109.5	31.90
			**50 °C**			
46	110.0	29.66	105.6	22.96	96.1	28.92
400	91.4<br>131.7	12.10<br>2.16	97.1	19.00	104.4	7.42
810	95.9<br>133.1	5.65<br>1.33	93.0	8.84	104.9	7.41
1340	103.4	3.52	101.6	5.39	97.4	4.90
2100	82.7	3.58	96.7	3.51	-	-
3300	86.5	2.26	99.5	3.40	90.2	3.22
4000	80.2	4.21	-	-	88.4	3.42
5000	-	-	-	-	77.2	3.02
			**55 °C**			
46	96.9<br>133.3	6.83<br>1.70	96.6	7.48	103.6 133.2	3.71<br>0.44
400	105.3	3.55	104.1	5.41	109.9	3.33
810	88.8	9.27	101.8	6.83	-	-
1340	89.1	8.27	-	-	84.8	4.12
2100	84.8	3.78	-	-	87.3	0.86
3300	-	-	85.9	4.73	-	-
4000	-	-	83.4	3.73	-	-
5000	-	-	-	-	-	-
			**60 °C**			
46	126.4	6.41	101.8	3.81	117.1<br>135.9	6.45<br>0.42
400	86.4	5.94	-	-	-	-
810	86.8	8.95	-	-	-	-
1340	83.3	3.85	-	-	-	-
2100	-	-	-	-	-	-
3300	-	-	-	-	-	-
4000	-	-	-	-	-	-
5000	-	-	-	-	-	-

Table S3. DSC results of melting behavior (T_m and ΔH_m) of samples.

	PLA		PLA/CC		PLA/CC/P	
**Time (h)**	**T_m (°C)**	**ΔH_m ($J{\cdot}g^{-1}$)**	**T_m (°C)**	**ΔH_m ($J{\cdot}g^{-1}$)**	**T_m (°C)**	**ΔH_m ($J{\cdot}g^{-1}$)**
0	146.7	−25.71	147.2	−17.25	144.8<br>150.4	−31.82
			**50 °C**			
46	145.5	−29.30	142.9	−22.65	148.6	−30.71
400	149.0	−36.67	143.4	−23.03	148.6	−31.24
810	149.5	−40.58	143.7	−23.84	149.3	−32.19
1340	148.5	−61.46	146.4	−23.44	147.9	−39.57
2100	141.4	−55.70	149.5	−25.86	150.4	−48.57
3300	129.3	−61.46	149.9	−26.83	140.0	−57.86
4000	124.1	−67.11	149.5	−28.54	129.5	−55.48
5000	108.9	−52.61	150.0	−40.39	121.8	−54.14
			**55 °C**			
46	148.0	−26.75	144.7	−21.04	148.4	−28.52
400	151.2	−39.69	145.1	−24.37	149.2	−34.56
810	141.3	−54.87	146.1	−26.69	149.3	−44.05
1340	132.5	−66.78	149.4	−27.25	138.9	−58.05
2100	128.2	−68.22	149.7	−42.60	131.0	−59.17
3300	110.4	−52.09	136.7	−65.03	112.0	−75.56
4000	91.5	−67.11	127.8	−65.19	76.6	−67.40
5000	69.4	−57.74	113.7	−54.95	75.6	−66.40
			**60 °C**			
46	149.3	−26.24	149.3	−20.42	145.0	−26,16
400	140.2	−62.77	140.2	−30.73	147.0	−52,70
810	133.8	−66.74	133.8	−38.77	138.1	−65,83
1340	124.7	−65.69	127.9	−64.41	125.9	−65,61
2100	118.0	−53.37	131.1	−73.39	117.1	−65,57
3300	82.1	−63.91	111.5	−49.79	81.5	−79,86
4000	72.1<br>86.5	−60.97	89.8	−69.66	85.7	−73,08
5000	81.4<br>108.1<br>123.7	−61.25<br>−1.90<br>−6.00	70.1<br>84.4	−56.40	58.8<br>82.2<br>110.5	−2.99<br>−67.93<br>−1.46
